# Overview of Electrochemical DNA Biosensors: New Approaches to Detect the Expression of Life

**DOI:** 10.3390/s90403122

**Published:** 2009-04-24

**Authors:** Stefano Cagnin, Marcelo Caraballo, Carlotta Guiducci, Paolo Martini, Marty Ross, Mark SantaAna, David Danley, Todd West, Gerolamo Lanfranchi

**Affiliations:** 1 CRIBI Biotechnology Centre and Department of Biology, University of Padova, via U. Bassi 58/B 35121 Padova, Italy; E-Mails: stefanoc@cribi.unipd.it; paolom@cribi.unipd.it; 2 CombiMatrix Corporation, 6500 Harbour Heights Pkwy, 301, Mukilteo, WA 98275, USA; E-Mails: mcaraballo@combimatrix.com; mross@combimatrix.com; msantaana@combimatrix.com; ddanley@combimatrix.com; twest@combimatrix.com; 3 DEIS Dipartimento di Elettronica, Informatica e Sistemistica, University of Bologna, Viale Risorgimento 2, 40136 Bologna, Italy; E-Mail: carlotta.guiducci@epfl.ch; 4 IBI-EPFL, Institute of Bioengineering, Ecole Polytechnique Federale de Lausanne, Station 15 CH-1015 Lausanne, Switzerland

**Keywords:** DNA chip, Electrochemical DNA detection, Biosensors, Microarray

## Abstract

DNA microarrays are an important tool with a variety of applications in gene expression studies, genotyping, pharmacogenomics, pathogen classification, drug discovery, sequencing and molecular diagnostics. They are having a strong impact in medical diagnostics for cancer, toxicology and infectious disease applications. A series of papers have been published describing DNA biochips as alternative to conventional microarray platforms to facilitate and ameliorate the signal readout. In this review, we will consider the different methods proposed for biochip construction, focusing on electrochemical detection of DNA. We also introduce a novel single-stranded DNA platform performing high-throughput SNP detection and gene expression profiling.

## 1. Introduction

DNA microarrays are the forefathers of DNA biosensors. They were born in response to the completion of a number of whole genome sequences to investigate the resulting large numbers of characterized genes. The power of this technology was demonstrated primarily by the work of Affymetrix [1–5] and Stanford University groups of Davis and Brown [6–12]. DNA microarrays are used to measure mRNA or miRNA expression [13–19], to characterize single nucleotide polymorphisms (SNPs) [19–23], to identify *in vivo* Transcription Factor (TF) binding sites [24–26] and as a diagnostic tool to determine chromosome deletion or amplification [27,28]. However, the size of samples and numerous preparative steps limit microarray studies in tissue-specific or cell-specific responses [19,29], or prevent them from delivering results in real-time. In spite of these limitations there are different approaches to study gene expression with very scarce sample sources derived, for example, from laser capture micro dissection approach [30–32]. These methods are based on RNA amplification [33,34], or signal amplification of detected fluorescence using tools such as dendrimers that, thanks to their chemical structure, allow the accumulation of many fluorescent molecules into the target[35], or enzymes that catalyze serial depositions of fluorophores after target-probe binding (tyramide signal amplification (TSA) method) [36].

DNA biosensors have the potential to overcome the limits of DNA microarrays by offering rapid and high sensitive analytical tools for genetic detection [37]. The most important challenges are: i) the integration of microelectronics to microchip-based nucleic acid technologies in a high scalable process; ii) the automation of the detection step and iii) the ability to perform direct signal transduction avoiding the images processing and statistical analysis, necessary in canonical DNA microarray workflow [38]. Potential applications of DNA biosensors include molecular diagnostics [39,40], pharmacogenomics [41,42], drug screening [43–45], medical diagnosis [46,47], food analysis [48–50], bioterrorism [51] and pollution [52–54] or environmental [55] monitoring. Recently, new generations of chips that can perform DNA sequencing have been developed accelerating biological and biomedical research in the genetic field [56]. These new technologies are based on cyclic-array sequencing and include the following commercial products: the 454 Genome Sequencer (Roche Applied Science), the Solexa (Illumina), the SOLiD platform (Applied Biosystems), the Polonator (Dover/Harvard) and the HeliScope Single Molecule Sequencer (Helicos). Array-based sequencing enables a much higher degree of parallelism than conventional capillary-based sequencing, but presents problems with long sequencing runs and accurate data fidelity [57].

In spite of the potential of biosensors and their wide application in research, only some chips have entered the clinical market. Among these are the glucose sensors that were leading the market until a few years ago: 6% of the Western world population is, in fact, affected by diabetes and would benefit from the availability of rapid, accurate and simple biosensor for glucose. Nowadays, however, there is a great demand for monitoring other molecules such us cholesterol, lactate, urea, creatine, that are very important markers for health care. The reason for this limited adoption of biosensors in the market is that many critical parameters, such as quality control and selection of testing parameters and control need to be improved. Moreover, new projected biosensors have to meet the need that were not accomplished by the existing analyzers and have to provide some distinct advantage, for example improved ease of use, faster response time and portability.

In this review we introduce the DNA microarray technique as a benchmark to compare DNA biosensors. We will discuss DNA biochips as an alternative to conventional microarray technology, considering different approaches that have been proposed to facilitate and ameliorate the signal readout and focusing on the electrochemical DNA signal hybridization detection. This approach is very useful for the biosensing of sequence-specific binding of DNA because of the high sensitivity and the rapid response. In the last part of this work we introduce a new single-stranded DNA microarray sensor, developed by CombiMatrix, capable of detecting the presence and measuring the abundance of thousands of different genes.

## 2. Conventional Microarrays

Conventional microarrays fall into the category of biosensors only in a general sense, but they represent a benchmark for DNA biosensor comparison. Molecular recognition events are based on nucleic acid hybridization events that are transduced into a detectable signal; usually fluorescence [58,59]. The hybridization is a peculiarity of single-stranded nucleic acid (DNA or RNA) thanks to the hydrogen bonds formed between adenine (A) and thymine (T), or guanine (G) and cytosine (C) bases in DNA, while in RNA, thymine is replaced by uracil (U). DNA microarrays are characterized by high-density probes (100 - 1 million DNA probes can be attached to a surface of 1 cm by 1 cm) linked to a solid surface to which labeled target hybridizes [19,60,61]. Probes could be PCR products (> 500 mer; cDNA microarrays) [7,8,62] or oligonucleotides (20 – 70 mer) [3,13] that are deposited onto the solid surface or directly synthesized onto the surface [63] (Table 1). Synthesized oligonucleotide sequences are a function of the knowledge of the genome of the studied organism. Today the sequencing of a complete genome is becoming an easier task thanks to the availability of new cyclic-array sequencers [57]. This second generation of sequencer uses a high degree of parallelism, spatially arraying DNA fragments to be sequenced, resulting in lower cost protocols. Today, multiple investigators are working on technologies for ultra-fast DNA sequencing. These are based on nanopore sequencing [64,65] or real-time monitoring of DNA polymerase activity [66,67]. In the first case nucleic acids are driven through a nanopore modulating the ionic current through the nanopore and allowing to the nanopore itself to work as a biosensor [64]; in the second case a zero-mode waveguides permit to detect the nucleotide incorporation during DNA polymerization in a zeptoliter-scale volume [66]. The cost reduction of DNA sequencing by massive sequence parallelization, is democratizing the knowledge of genomic information of different organisms (e.g. economically important like *Vitis vinifera* [68]) and opening the door to functional genomics studies by DNA microarrays to any organism or biological condition.

Different companies have developed different strategies to produce their DNA microarray using phosphoramidite chemistry and reactive protective groups in the last added nucleotide of the growing DNA oligonucleotide. Protective groups prevent unwanted side reactions and force the formation of the desired oligonucleotide sequence during synthesis. Affymetrix, Nimblegen (Roche) and Febit platforms use the light to activate particular chip sites but extend the oligonucleotide length with photolithography masks in the first case [5], or micromirrors in the second and third cases [69–71]. The Agilent technology uses ink-jet technology to spot the amidites and employs a flooded chemical deprotection [72] while CombiMatrix uses an addressable electrode array for the production of acid at sufficient concentration to allow deprotection process and to permit the oligonucleotide synthesis [73]. Nanogen, a company that has been on the market since 1997, developed a microelectronic array used to influence DNA transport, concentration and hybridization changing physical parameters like DC current, voltage, solution conductivity and buffer species (APEX technology) [74] (Table 1). Illumina and Motorola have developed novel 3D microarrays. Illumina combines the association of microbeads linked to specific probes and an array of microwells that could accommodate one bead per well, allowing the organization of an ordered array [75–77] while Motorola has developed a three-dimensional matrix that enables the attachment of biomolecules to the slides.

**Figure 1.**
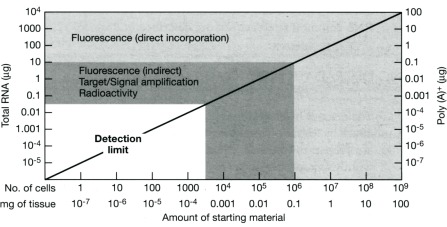
Description of the RNA amount utilized in the different microarray labeling techniques. The RNA amount is related to the cells number or tissue weight with a detection limit of 1000 cells. Direct incorporation of fluorescent nucleotides into the cDNA can be used to examine expression of samples with 10 μg of total RNA while indirect incorporation of fluorescent nucleotides is used with samples presenting total RNA concentration between 10 μg to 50 ng. Described detection limits do not preclude the use of the microarray to perform genome wide studies of biopsies or histological samples. Image reproduced from Duggan *et al*.[29].

Detection of the hybridized targets in microarrays is related to the labeling process of the target itself. It could be coupled to RNA linear amplification [33,34], depending on the quantity of the starting material, or used as a direct or indirect method to incorporate the fluorescence in the synthesized target[78] (Figure 1). Nowadays microarray sensitivity ranges from 50 fM to 10 pM of mRNA target that is present in the solution. The differences are expressed in a relative (ratio-based) mode [19], but recently Carter [79] developed a method based on spike-in and on the generation of dose/signal graphs to obtain absolute expression measurements (proportional to transcript copy number).

## 3. DNA Biosensors

**Figure 2.**
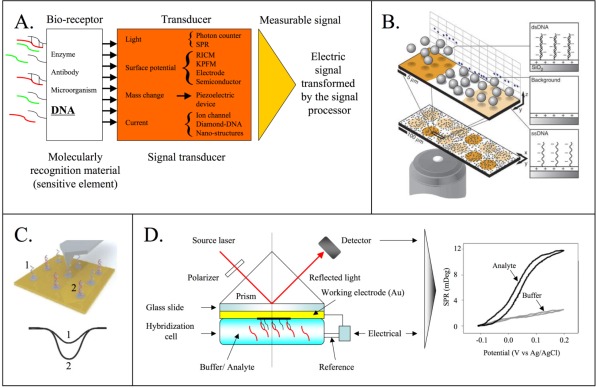
A). Scheme of a biosensor. The three main components of a biosensor are evidenced: the sensitive element (white box), the transducer with a list of different transduction methods (orange box) and the signal processor (yellow triangle) that displays the transformed signal in a user-friendly way. B). RICM method description. A suspension of negatively charged silica microspheres is gravitationally sedimented over a microarray surface allowing the electrostatic readout of microarray. The positions and motions of a population of microspheres are used to image the surface charge of the microarray and detect hybridization. This is caused by the higher negative charge of the areas displaying double-stranded DNA in comparison to those displaying ssDNA, and both contrast with the positively charged background. Image reproduced from Clack *et al*. [90] C). Schematic view of DNA probe in single (1) and double stranded (2) conformation (hybridized to target molecules) scanned by the KPFM method. Bottom image represents a typical KPFM response in which electrostatic potential is plotted against surface position. Point 1 and 2 evidence the different responses of the surface potential according to the absence (1) or presence (2) of hybridization with target. D). EC-SPR scheme. The combination of SPR and electrochemical techniques allows obtaining new insight in the interfacial recognition process. The cyclic voltammetry and the simultaneous measure of the SPR angle show a sigmoidal change between the oxidized and the reduced state of the analyte. The cyclic voltammogram and SPR response in the absence of the analyte are shown, for comparison.

Biosensors are devices that combine a biological component with a detector component. Biosensors consist of three parts: i) the sensitive elements (biologically-derived material), ii) the transducer or detector element that transforms the detected signal in a readable and quantified output and iii) the signal processor, that display the transformed signal in a user-friendly way (Figure 2A). In DNA biosensors the sensitive element is generally composed by single stranded DNA (ssDNA) molecules that allow the hybridization of complementary single-stranded molecules [63,80–91]. Different methods can be used to transduce these hybridization signals including: a) optical transducers that are based on fiber optics [77,92], reflection interference contrast microscopy (RICM)[90], surface plasmon resonance (SPR) [93,94] or Raman spectroscopy [95–97], b) electrochemical transduction [80–84, 86,88,89,98] or electrical transduction (e.g. integrated-circuit (IC) biochip [99] in association with molecular beacon (MB) [100]), and c) piezoelectric transduction (measurement of changes in mass) [85,101–106] (Table 2).

Optical methods are the most frequently used in the detection of analytes because of their simple and straightforward use [59,61]. A variety of optical methods are based on target labeling with radioisotopes, fluorophores and UV-absorbing molecules. Fluorescence is an event occurring to molecules like polyaromatic hydrocarbons or heterocycles when they absorb light. They change their energy level if excited by light and decay from the excited energy level by emitting fluorescent light. Although the fluorescent approach based on fluorescence is simple, it is influenced by the environment (solvents, pH and conjugation to nucleic acids). Moreover fluorescent dyes could be toxic molecules for the user. For example, UV-absorbing compounds like ethidium bromide, a standard fluorescent dye for staining DNA, is known to be mutagenic and carcinogenic. Further disadvantages of the fluorescence–based approaches are also the instrumentations used for signal reading that are not easily transportable and generally expensive. Different optical approaches were developed to overcome the limits of fluorescence and to avoid target tagging (i.e. labeling of the DNA). These methods are based on Raman spectroscopy [95,96], RICM [90] or SPR [93,94]. The first method provides femtomolar sensitivity and multiplexed detection of DNA and RNA targets with single nucleotide polymorphisms [97]. The method is based on photons scattering when incident light encounters a molecule. Also in this detection process is necessary to label the target with the Raman-active dyes. Only RICM and SPR are genuine label-free optical methods. In the first case the association of negatively charged microbeads with the reflection interference contrast microscopy (RICM) produces an image of the hybridized or not hybridized targets to the respective probes (Figure 2B). The limit of this technique is not diffraction, but the particle position resolution and the concentration of the particle in the solution. Clack and collaborators, using 30-mer capturing sequence, described a detection limit of the RICM method as 50 pM, but similar limits as seen with fluorescence detection (1 – 5 pM) may be anticipated, using electrostatic readout in optimal substrate and hybridization conditions [90]. To scan the surface potential Sinensky and Belcher [107] evidenced the advantages of Kelvin probe force microscopy (KPFM). KPFM is a non-contact variant of atomic force microscopy (AFM) based on the measurement of the electrostatic forces between the small AFM tip and the sample. Since DNA strands are negatively charged, it is possible to measure the presence of a specific bound target on a DNA modified surface avoiding the labeling step (Figure 2C). Sienencky and Belcher demonstrated a sensitivity of 50 nM, that is lower than the sensitivity of the RICM technique, and a resolution of < 10 nm [107].

SPR is an optical-electrical phenomenon arising from the interaction of light with a metal surface, making the detection of the presence of a biopolymer on chemically modified gold surface possible. The basic principle involved is the change in the local index of refraction upon adsorption of light. The optical phenomenon is linearly related with the mass concentration adsorbed onto the metal film. The BIAcore 3000 instrument integrates SPR technology with a microfluidics system to monitor molecular interactions at real-time molecular interactions at concentrations ranging from pM to mM. The BIAcore instrument was used in virology applications to detect HIV-1 genomic sequences [108] demonstrating the possibility to use it in an automated diagnostic system. The SPR technique is a label free, high throughput and scalable method in array format. It was used by Goodrich and collaborators to detect multiple DNA targets at a concentration of 10 fM on a single chip [93].

SPR could be used in association with electrochemistry (EC-SPR) where the thin metal film on the substrate is used not only to excite surface plasmons, but also acts as a working electrode for electrochemical detection or control [109,110]. With the EC-SPR configuration is possible to simultaneously obtain information about the electrochemical and optical properties of films with thicknesses in the nanometer range (Figure 2D). Georgiadis *et al*. and Heaton *et al*. monitored the *in situ* hybridization of DNA in the presence of different electrochemical fields [109,110].

The mass of the absorbed molecules is the measured parameter also in the piezoelectric sensors. The method used is based on the change in oscillating frequency resulting from the increase in mass on the crystal surface, which accompanies the hybridization. A quartz crystal microbalance affinity biosensor was used by Mannelli and collaborators to detect genetically modified organisms [105] and recently chemically modified piezoelectrodes were utilized to develop a biosensor for the determination of genetically modified soybean [106]. This approach is used for the identification of genetically modified organisms (GMOs) because the production of GM crops is increasing and there is a growing requirement for methodologies that allow the accurate and easy determination of the content of GMOs [111].

## 4. Electrochemical/Electrical DNA Biosensors

In order to reduce the size of the instrumentation needed in the DNA detection and the costs incurred for this purpose, DNA chips that can perform target detection with an electrical signal have been proposed [61,140,141]. In fact, the miniaturization of electrochemical devices and technology improvements make them excellent tools for DNA diagnosis.

The immobilization of the ssDNA onto the transducer surface plays an important role in the performance of the biosensors because the surface modification technique must be compatible with the related sensing methodology [91]. Various methods have been developed to attach the DNA probe to the solid surface of biosensors: the self assembling monolayer (SAM) on gold electrodes [142–144], biotinylated DNA probes attached through biotin-avidin interaction on electrode surface [145] or electro polymerization that produces probes of different length [73]. A new challenge is the development of dynamic surfaces with the ability of tuning their biochemical functionality. Moore *et al*. [146] have proposed a thiol-functionalized surface to which molecules or probes could be attached by a disulphide bridge that, after the detection process, could be chemically or electrically renewed and reused.

Electrical detection mode was developed for detection of both label-free and labeled DNA targets. The first method allows a direct transduction translating the recognition behavior in a readable signal in real time mode performing kinetic measures [81]. In contrast, labeling approaches involve the detection of a marker, associated with the duplex formation. The labeling step enhances the sensitivity and the selectivity, but also increases the time, complexity and cost of measurement.

### 4.1. Nano-Objects for the Electrochemical Biosensors

**Figure 3.**
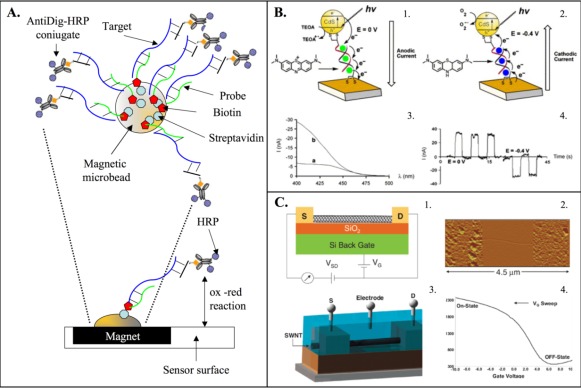
A). Schematic representation of the electrochemical strategy used for the detection of food pathogens by Lermo *et al*. [128]. Biotinilated probe is immobilized onto magnetic beads and hybridized with the target. Enzymatic labeling, magnetic capture of the modified magnetic beads by the magneto electrode and chronoamperometric determination are common steps for this strategy. B). Photochemical detection of DNA hybridization. The exposure of the CdS nanoparticles to visible blue light gives rise to a directionally electroswitchable photochemical current (1 and 2) between the nanoparticle and gold electrode. (3) Photocurrent action spectra generated in the CdS nanoparticles DNA system: in the absence (a) and in the presence (b) of methylene blue. (4) Electrochemically switched anodic and cathodic photocurrents generated in the Cd nanoparticles DNA in the presence of methylene blue generated at 0 and −0.4V. Photocurrents were generated upon irradiation at λ = 420 nm. Image reproduced from Willner *et al*. [130]. C). Carbon nanotubes field effect transistor. (1) A NTFET device composed of an isolated single-walled carbon nanotubes (SWNT) between source (S) and drain (D) electrodes on top of a SiO_2_ substrate with an underlying Si gate electrode. (2) An atomic force microscope (AFM) image of the NTFET device illustrated in part 1. (3) A liquid gated NTFET, where the electrochemical potential of the solution is controlled with a gate electrode. (4) NTFET transistor characteristic showing the source–drain conductance versus gate voltage (G–V_G_) curve obtained by sweeping the gate voltage from +10 to −10 V at a constant S–D bias voltage (V_SD_) of 0.05 V using a NTFET with a random network of SWNTs between interdigitated Ti/Au electrodes on a SiO_2_ insulated Si back gate. Image reproduced from Kauffman *et al*. [118].

Nanomaterials have facilited the development of ultrasensitive electrochemical biosensors because of their high surface area, favorable electronic properties and electrocatalytic activity [147]. Moreover, they show good biocompatibility due to their nanometer size and specific physicochemical characteristics. Nanoscale materials include nanoparticles, nanowires, nanoneedles, nanosheets, nanotubes, nanorods, nanobelts, etc. The use of magnetic micorbeads has also gained popularity. They are used to fish targets that are homologous to the probes linked to the bead surface and to concentrate the hybridized target by bead precipitation. This strategy was utilized by Lermo and co-workers [128] for the electrochemical detection of pathogens in food allowing the detection of DNA at femtomolar level (Figure 3A). Gold nanoparticles was used by Park *et al*. [112] in a typical sandwich approach to close the electrical connection between two flanking microelectrodes demonstrating a sensitivity of ≅ 5 × 10^−13^ M in target DNA. In this technique DNA probes have been deposited between separated microelectrodes to discriminate between positive and negative hybridization signals, basing the circuit resistance among the electrodes. Resistance is modulated by the presence of the gold nanoparticles that detect the presence of hybridized target.

Gold nanoparticles are also used in the pencil graphite electrode DNA sensor onto which probe strands are immobilized. Hybridization is detected electrochemically with the appearance of a characteristic gold-oxidation signal with a detection limit of 0.8 femtomoles of DNA, thanks to the large electrode surface and the high number of oxidizable gold atoms in each nanoparticle [129]. Nanoparticles are also suitable for the photochemical detection of DNA hybridization. Willner *et al*. [130] used CdS nanoparticles in DNA hybrid system associated with an electrode relying on the exposure of the CdS nanoparticles to visible blue light which gave rise to a photochemical current between the nanoparticle and gold electrode (Figure 3B).

Nowadays other nano-objects such as nanowires [131] and carbon nanotubes [132,133] have received increasing attention. Nanowires represent a class of inorganic materials that are surface-passivated by thin oxide layer and serves as electrodes or can interconnect between micro- and nanoelectronic devices. Carbon nanotubes exhibit properties such as robustness, enormous specific surface area and large-scale arrayability, but the extreme sensitivity of nanowires and nanotubes field-effect sensors (Figure 3C) is balanced by their sensitivity to impurities and other ionic species in analyte solution. As a result, low ionic strength buffer is quite often necessary, and studies on sensing mechanism have been proven to be difficult [134].

### 4.2. Label-Free Electrochemical DNA Detection

As mentioned earlier, label-free DNA detection involves the measurement of physiochemical changes occurring on the surface of the transducer device due to the DNA hybridization. Comparing with label-free optical and physical transduction (SPR, quartz crystal microbalance, KPFM and RICM) electrical transduction is cheaper, portable, independent of sample turbidity, easily miniaturizable and more compatible with nanotechnology. The earliest label-free approach was based on the intrinsic electroactivity of DNA purine bases. 50 years ago Palecek *et al*. [148] developed methods to discriminate ssDNA versus dsDNA through direct DNA reduction. The direct oxidation of DNA requires relatively high potentials causing significant background currents. To improve signal-to-noise ratio a two-step strategy was proposed for, first, capturing target and then detecting the oxidation process [135]. According to this process, hybridized target to probes linked to magnetic beads is purified using the beads itself and then is analyzed using adsorption stripping voltammetry after the depurinization. 40 femtomoles of substrate have been detected by this assay. Electrochemical assay sensitivity is therefore comparable to SPR [109,110] as described in the DNA biosensors paragraph.

Starting from the idea of Aviram and Ratner [149], who used organic molecules as electronic components, different strategies have been developed to detect DNA hybridization in transistor devices in a label-free mode. In 2004 Kim *et al*. [113] fabricated a field effect transistor (FET)-type DNA charge sensor based on standard complementary metal oxide semiconductor (CMOS) technology which can detect the DNA probe immobilization. Detection occurs by sensing the variation of drain current due to the change in charge distribution at the interface induced by DNA binding. A FET-type charge sensor is a semiconductor sensor that measures the change of the oxide/electrolyte interface potential caused by DNA probe immobilization and target detection on the gate metal, based on the field effect mechanism of MOSFET [88,116]. This structure was utilized by Bandiera *et al*. [81] to make a fully electronic sensor for the measurement of the DNA molecules kinetic hybridization since with this sensor configuration it is possible to measure the charge variation on the detector dynamically during time. They demonstrated that long DNA strands have slower hybridization kinetics than short DNA strands. This is probably related to different movement ability and steric constraints of DNA in solution. FET devices are attractive structures in association with nanomaterials such as carbon nanotubes, described in the previous paragraph. About ten years ago unique devices based on carbon nanotube field-effect transistor (NTFET) technology appeared [117,136] with the conduction channel formed by carbon nanotubes. The small diameter and relatively long length (μm) of single-walled carbon nanotubes (SWNTs) allow them to probe molecular systems on a local scale by directly connecting to individual or small assemblies of molecules. These characteristics of SWNT based NTFETs create unique platforms for studying molecular systems with unsurpassed sensitivity (≅500 pg/mL of target DNA) [115,118] (Figure 3C). The measure of potential surface by FET devices is very attractive because the transduction device integrates the sensing element and because of the possibility of system miniaturization. However, performance remains a function of solution characteristics, the probe immobilization techniques and the thickness of the insulator oxide.

**Figure 4.**
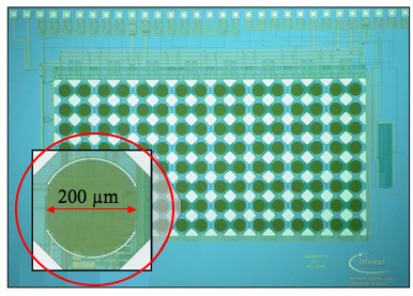
Microphotograph of a fully digital 16 × 8 sensor array (source: Infineon Technologies AG [150]). The chip is 4.15 mm × 5.65 mm; sensor pitch is 250 microns. Each site is an interdigitated gold electrode couple exposed to the solution.

Capacitive measurements were used in the work of Stagni and collaborators [114] who demonstrated the ability of a CMOS DNA based chip to detect the hybridization in a label-free DNA detection. The CMOS chip with 128 sites is shown in Figure 4. This work is based on circuits that measure the electrodes’ interface electrochemical impendance. From the electrical perspective, the interface between the electrodes and the solution is characterized by capacitive and resistive parameters sensitive to the electrode surface state. The presence of single strand or double strand (hybridized) DNA affects the interface electrical parameters. Hybridization decreases the interface capacitance of the gold electrodes covered with the specific probe [119]. Guiducci *et al*. [86] demonstrated the possibility a) to detect DNA hybridization in a two-gold-electrode system without using a reference electrode and b) to integrate the technology in an integrated silicon chip of gold microelectrodes on a 2 × 10^3^ μm^2^ surface [120]. The ability to use this technique with two-gold-electrode system instead of three electrodes setup make it possible to develop a cost-effective fully integrated design. In fact, the reference electrodes are problematic, being subjected to aging and requiring specific storage and regeneration procedures.

### 4.3. Indirect Electrochemical DNA Detection

Even if label-free DNA detection simplifies the readout and reduces time and costs of analysis, it does not reach the same sensitivity of the label (indirect) DNA detection. The indirect method permits sensitivity at the atomole level in term of concentration of the DNA target [121]. Indirect methods also require mediators that facilitate electron transfer between them and the electrode. Redox mediators are small size compounds that enable the reversible exchange of electrons with the electrode. The most used electron mediators are ferrocene, K_3_Fe[(CN)]_6_^3−/4−^, Ru(bpy)_3_^3+/2+^, Os(bpy)_3_^3+/2+^ and Methylene Blue. As mentioned in the previous paragraph, nanomaterials could also be used as indirect electrochemical sensors. For example, metal nanoparticles represent a large redox reservoir [129].

The electron mediators are used: i) in reactions to oxidize directly DNA, ii) to avoid the modification of the target strand, and iii) in reactions modulated by enzymes linked to the target strand. In the first case for example, Yang and Thorp [122], using electrocatalysis by Ru(bpy)_3_^3+/2+^ and the oxidation of the immobilized guanines, were able to discriminate the genomic expansion of the triplet repeat sequences 5′-(CTG)n and 5′-(CGG). These expansions are responsible for the myotonic dystrophy and fragile X syndrome respectively. To avoid the target modification Umek *et al*. [123] performed a three-component sandwich assay, in which the redox label was attached to a synthetic sequence specifically designed to bind an overhang portion of the probe target complex.

Target enzyme markers are an attractive, well documented strategy for the time-controlled production of redox species. For instance, alkaline phosphatase (AIP) [124,125], glucose oxidase (GOx) [126] and horseradish peroxidase (HRP) [98,127] have been used for the fabrication of electrochemical sensors. Today, an ideal biosensor is required to be not only miniaturized and cost-effective, but also capable of simultaneous detection of multiple analytes. To this purpose the CombiMatrix company developed a DNA sensor chip detecting 90K with fluorescence method and 12K in both fluorescence and electrochemical method.

### 4.4. CombiMatrix Chip: A High Throughput DNA Sensor

**Figure 5.**
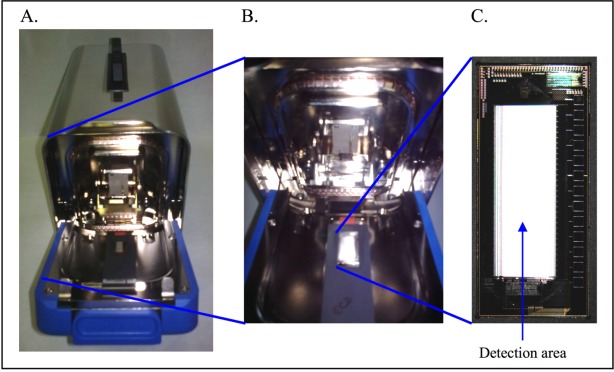
A. ElectraSense reader. B. Enlargement of the chip housing. C. Enlargement of the silicon chip with complementary metal oxide semiconductor (CMOS) circuitry. The central detection area shows 12k electrodes. Diameter of each electrode is 44 μm.

The CombiMatrix 12K ElectraSense^®^ microarray is a silicon chip with complementary metal oxide semiconductor (CMOS) circuitry (Figure 5). This circuitry is addressed through pogo pin connectors to thirteen metal pads at the side of electrode field. On-chip logic and Windows^®^ software control the circuitry to address each of 12,544 electrodes individually or in predefined groups. The microarray becomes a highly multiplexed transducer where electrical signals drive chemical reactions or chemical reactions create electrical signals at each electrode.

As a multiplex sensor, the specificity of the ElectraSense microarray is dictated by the capture molecules on each electrode. Maurer *et al*. [73] described the use of electrochemistry to generate specific DNA probes on each electrode to create a custom array for genomic testing. Asai and collaborators first reported on using an early version of the ElectraSense microarray for selecting and mutating aptamer sequences to improve binding to resorufin [151,152]. More recently, Knight *et al*. applied a sophisticated *in silico* modeling approach for creating high binding aptamers to allophycocyanin [153]. Both groups of investigators used the customizable feature of the microarray to iteratively change (mutate) a few nucleic acids on the aptamers to determine the strongest binding sequences. Based on the electrochemical synthesis of unique DNA sequences at each electrode, the ElectraSense microarray can serve as detector for specific oligonucleotide binding and binding by other molecules as well.

Detection of molecular binding on the microarray can be measured using fluorescence or electrochemical detection (ECD). For measuring oligonucleotide binding using ECD, commercially available reagents that are used for ELISA assays perform very well, including avidin-HRP, tetramethylbenzidine and hydrogen peroxide. Ghindilis *et al*. [127] compared the efficiencies of fluorescence detection and ECD using a spike-in experiment and determined that ECD had a lower limit of detection of 0.75 pM while fluorescence had a lower limit of 1.5 pM. The average correlation coefficient between fluorescence and ECD in these studies was 0.94.

Given these indices of performance, fluorescence detection and ECD on CombiMatrix 12K microarrays are comparable; however, the instrumentation for ECD is considerably less complex and far more rugged. The 12K ElectraSense microarray can be read in less than 15 seconds using a single 9 cm by 6 cm electronics board that is powered through a PC USB 2.0 port (Figure 5). This reduction in size and complexity allows the array to be integrated into fluidic cartridges without concern for optical paths and instrument stability. By modifications to the electronics board, a potentiostat can be connected to individual electrodes or groups of electrodes for cyclic voltammetry and impedance spectroscopy. Redox molecules, such as ferrocene, Methylene Blue and ferri/ferrocyanide can be used for making these measurements on the ElectroSense microarray. Using a potentiostat to measure molecular interactions on the array reduces the capacity of the microarray to multiplex; however, this loss is traded against reducing the number of reagents required for detection. Using different approaches to detection on a single platform provides orthogonal assay validation and allows the investigator to quantify and compare performance against expenditure of resources.

### 4.5. Charge Transport by DNA

An alternative approach for the electrochemical DNA detection is based on DNA-mediated charge transport. Redox-active reporter molecules, non-covalently associated with double helix, have been successfully used for electrochemically based DNA analysis. Milan and Mikkelsen [137] demonstrated the use of electroactive hybridization indicators in a reusable sequence-selective biosensor for DNA. Steel *et al*. [138] in 1998 developed and measured the surface density of DNA on gold electrode. The number of nucleotide phosphate residues was calculated from the amount of cationic redox marker measured at the electrode surface.

A different approach was based on intercalating redox probe molecules into the double-strand DNA structure. These intercalated molecules can monitor perturbations in base stacking[154] and thus discriminate between perfect and imperfect hybridized target. This assay is well suited for mutational analysis [139]. Briefly, after the formation of DNA duplex on gold surface and the treatment with a redox active intercalator a reporter molecule could be electrochemically reduced by DNA-mediated charge transport. The detection of mismatches does not depend on the thermodynamic destabilization, but by the ability of the electrons to flow along the double-strand DNA.

## 5. Conclusions

The development of biochips has a long history, starting with the first portable glass pH electrode (1922) [155], and is continuing today. DNA biosensors and microarrays are of considerable recent interest due to their tremendous promise for obtaining sequence-specific information in a faster, simpler and cheaper manner compared to traditional hybridization assays based on RNA radiolabeling [156]. From the first description of the structure of the double strand DNA, by Watson and Crick (1953), few scientific areas have witnessed dramatic changes of the magnitude observed recently in DNA diagnostics [157]. The rapid technological advances of the biochemistry and semiconductor fields in the 1980s led to the large-scale development of biochips in the 1990s. In fact biosensors are becoming one of the most popular scientific areas at the intersection of the biological and the engineering sciences [158]. The traditional separation between transducers and bioreceptors is being replaced by an integrative approach. e.g. STMicroelectronics, a silicon chip company has developed a silicon chip integrating microfluidic handling, a miniaturized PCR reactor connected to a custom microarray [159].

This work reviewed the forefathers of DNA biosensors that are used in laboratories worldwide to detect differentially expressed genes in atherosclerosis [13], leukemia [160], skeletal muscle dystrophies [161,162] and in many other pathologies [163,164]. Apart their diffusion DNA microarrays are difficult to use, require specialized operators and complex bioinformatics analysis [19]. As such they are not classical biosensors. Researchers have utilized several approaches, herein described, to respond to the demand for user-friendly, portable, sensitive, miniaturized and low cost DNA sensors to support or substitute DNA microarrays.

All of the detection methods described in this review have caveats, but those based on electrochemistry are particularly interesting because their sensitivity (fM in association with nanoparticles [129]), and the opportunity to miniaturize the technology. Nano-objects play an important role in the development of electrochemical DNA sensors. Their nanometer size makes them highly reactive and represents the ultimate miniaturization level for DNA sensors.

The attractive properties of electrochemical devices are extremely promising for improving the efficiency of diagnostic testing and therapy monitoring even more today with the construction of very large multiplexed array: the CombiMatrix sensor. Future biosensors will require the development of new reliable devices or the improvement of the existing ones for use by non-specialized personnel without compromising accuracy and reliability. Compact and portable devices will constitute another future area of multidisciplinary research on sensors.

## Figures and Tables

**Table 1.** Types of oligonucleotide and cDNA microarrays.

**Company name**	**Spotting method**	**Probe synthesis**	**Probe characteristics**	**Printing technique**	**Uptake volume (μL)**	**Dispense volume/Probe length**	**Spot diameter (μm)**
Affymetrix	Non contact	*In situ* synthesis	Oligonucleotide	Photolitography	NA	NA/20 – 25 mer	6.3
Nimblegen	Non contact	*In situ* synthesis	Oligonucleotide	Micromirror	NA	NA/50 – 75 mer	16
Febit	Non contact	*In situ* synthesis	Oligonucleotide	Micromirror	NA	NA/30 – 60 mer	24 – 72
Agilent technologies	Non contact	*In situ* synthesis	Oligonucleotide	Ink-jet	NA	NA/60 mer	60
CombiMatrix	Non contact	*In situ* synthesis	Oligonucleotide	Addressable electrode	NA	NA/35 −50 mer	25
Nanogen	Non contact	*In vitro* synthesis and electronic delivery	Oligonucleotide	Addressable electrode	NA	NA/Not specific length	80
Illumina	Non contact	NA	Oligonucleotide	Micro-beads	NA	NA/NA	3
“ArrayIt” TeleChem International Inc.	Contact	*In vitro* synthesis and spotting	Oligonucleotide	Printing tips	0.25	0.6 nL/NA	60 – 360
Eppendrof	Contact	*In vitro* synthesis and spotting	NA	Printing tips	0.25	0.6 nL/NA	60 – 360
Ocimum Biosolutions	Contact	*In vitro* synthesis and spotting	Oligonucleotide	Printing tips	0.25	0.6 nL/NA	60 – 360
Greiner Bio-One	Contact	*In vitro* synthesis and spotting	NA	Printing tips	0.25	0.6 nL/NA	60 – 360
SurModics (CodeLink array)	Non contact	*In vitro* synthesis and spotting	Oligonucleotide	NA	5 – 10	100 pL/ 30 mer	45 – 160
Academic/Universities	Contact/Non contact	*In vitro* synthesis and spotting	Oligonucleotide/cDNA	Printing tips/syringe solenoid or ink-jet	0.25/5 – 10	0.6 nl/ 100 pL 35 – 70 mer/ > 500 mer	60 – 360/120 – 500

NA: not available

**Table 2.** Types of DNA biosensors.

**Type**	**Transducer**	**Advantage/Disadvantages**	**Description**
Optical fiber	Fiber Optics	Sensitivity of optical approaches/Costly equipment and not portable	Fluorescence from labeled target is collected from the fibre waveguide [77,92]
Optical apparatus/Surface potential microscopy	Reflection interference contrast microscopy (RICM)	High sensitivity below water, dynamic image processing, rapid measurements/ Instrument required, not portable	RICM: a microinterferometric technique to measure absolute optical distances between transparent planar substrates and hard or soft surfaces such as colloidal beads or artificial and biological membranes, which hover over the substrate [90]
KPFM/Surface potential microscopy	Cantilever of AFM instrument	Accuracy of the measurement/scanning speed	Measure local variance in the surface potential [107]
Resonant minor (BIAcore)	Surface plasmon resonance (SPR)/EC-SPR	High sensitive/ Susceptibility to interference	Changes in the refractivity index [93,94,108] or associated with electrochemistry (EC-SPR) [109,110]
SERG probes	Raman spectroscopy	Spectra can be collected from a very small volume/ sensitive and highly optimized instrumentation	Plasmonics-based spectroscopic technique [95–97]
Diamond nanowires	Diamond	Fast/ High cost, buffered solution may interfere	Electrons from diamond substrate can flow along the DNA. Conductivity changes with ssDNA or ds DNA [89]
Active electrode/transistor surface/nano-structures	Electrodes/ Transistors/Nano-structures	Fast, relatively low cost/ Buffered solution may interfere	Analytes are involved in the reaction on the active electrode surface. The charge produced create a measured potential [80–84,86,88,98,112–139]
Opto-electronic photodiode	IC biochip in association with molecular beacon (MB)	Fast/ Dependent on fluorescence (bleaching)	Fluorescence of hybridized MB is collected and detected in miniaturized detection biochip[100]
Current across the channel	Ion channel	High-troughput/ Not well studied, buffered solution may interfere	Transmembrane voltage permit to draw DNA or RNA molecules through the open ion channel [64,65]
Quartz crystals/cantilever	Piezoelectric transducer	High sensitive, fast/ Sensitivity up to one cell has not been demonstrated	Changing in frequency of quartz crystals oscillation or cantilever deformation [85,101–106]
